# Population differences in completeness and reliability of Dutch COVID-19 registry data: The influence of testing policy, test readiness, and registration policy from an epidemiological perspective

**DOI:** 10.1007/s12508-023-00377-y

**Published:** 2023-03-03

**Authors:** Babette van Deursen, Ester A. L. de Jonge

**Affiliations:** 1 Department of Infectious Disease Control, Municipal Health Service (GGD) Rotterdam-Rijnmond, Rotterdam, The Netherlands; 2 Team Information, Registration & Research, Municipal Health Service (GGD) Utrecht region, Utrecht, The Netherlands; 3 cluster Direction, Research & Consulting, Municipal Health Service (GGD) South-Holland South, Dordrecht, The Netherlands; 4 Centre of Expertise Health Innovation, Lectorate Data Science, The Hague University of Applied Sciences, The Hague, The Netherlands

**Keywords:** Data quality, Population differences, COVID-19 registration, Data driven policy

## Abstract

During the COVID-19 pandemic, the bidirectional relationship between policy and data reliability has been a challenge for researchers of the local municipal health services. Policy decisions on population specific test locations and selective registration of negative test results led to population differences in data quality. This hampered the calculation of reliable population specific infection rates needed to develop proper data driven public health policy.

## Introduction to the problem

Over the past decade, Dutch municipalities and their public health services (i.e., the Dutch Municipal Health Services) have been experimenting with data-driven policy development and decision-making. During the COVID-19 pandemic, the growing role of data in governmental decision-making became visible to the general public as well. The numbers of positive test results, hospital admissions and deceased individuals were reported by the national news media on a daily basis. In addition, the use of more complex concepts, such as the reproduction number, were no longer exclusively used by scientists but became a regular part of the national press conferences during the pandemic. The use of terms such as ‘road maps’ and ‘signal values’ implies data reliability is high. However, in practice, data reliability is not always self-evident. The Dutch public debate focused on relevance of public health data for decision-making and policy development. In this article, we illustrate the bidirectionality of this relationship by showing how political decision-making and policy influenced data quality of the Dutch COVID-19 registries. We use data from a case study of three municipal health services in the southwest of the Netherlands. This study was initially designed to determine population differences in SARS-CoV2 infection rates [1].

Initially, variation in data completeness and quality mainly impacted the work of epidemiologists and other public health researchers serving local governments, as they were responsible for providing reliable information to policymakers. On the one hand, the political pressure to quickly provide reliable information on, for example, compliance with the test policy or infection risk among specific population groups was high. On the other hand, researchers could not influence the data collection process and the corresponding reliability of these data. For public health researchers, this created some tension between their data responsibilities and their ability to influence data.

## Aim and case study description

The primary aim of this article is to illustrate how the interaction between decisions of governmental institutions and decisions of individual citizens affected the quality of COVID-19 registry data. By data quality, we refer to a combination of completeness of the source data and reliability and relevance of the information derived from these data. Based on our findings, we will formulate a few recommendations that could contribute to improved quality of registry data in a potential future health crisis.

The initial research goal of our case study was to determine population differences in the prevalence of diagnosed COVID-19 in the southwest of the Netherlands, with a focus on living and labour conditions. Therefore, COVID-19 registry data of three municipal health services were merged with microdata of Statistics Netherlands (*Centraal Bureau voor de Statistiek*) [2] at an individual level. According to the Statistics Netherlands Act, Statistics Netherlands is tasked to perform statistical research commissioned by the Dutch government for practice, policy and research purposes and publish the statistical data compiled on the basis of such research. The COVID-19 data consisted of three main indicators: (1) the number of visits to the test facilities of these municipal health services, (2) the positive, negative or immeasurable test results obtained in these test facilities, and (3) the positive test results obtained at alternative test locations. Test facilities of municipal health services will be further referred to as ‘governmental test facilities’ in this article. Alternative test facilities included hospitals, general practices, nursing homes as well as commercial test locations. Microdata consisted of various indicators reflecting a subject’s socio-economic position (SEP), demography, and living and labour conditions.

Datasets merged at the individual level can reveal different details of information than datasets merged at an aggregated level, such as district level. This method contributes to the prevention of incorrect extrapolation of associations present at the aggregated level to the level of the individual. In epidemiology, this phenomenon is referred to as ‘ecological bias’ [3]. In our case study, data sets merged at the individual level provided an opportunity to identify characteristics of an individual associated with a higher infection rate.

Ecological bias could have occurred when residents of one district were at a higher risk of SARS-CoV2 infection than residents of another district due other factors than the determinants studied. Determinants studied in our case study were SEP, demographics, and living and labour conditions. Statistics Netherlands is highly motivated to preventing this type of bias. Therefore CBS offers scientists the opportunity to reuse its microdata at the individual level and pseudonymise merged datasets. The study period ranged from June 2020 to April 2021.

### Details of registration and testing policy relevant to case study

**Fig. 1:**
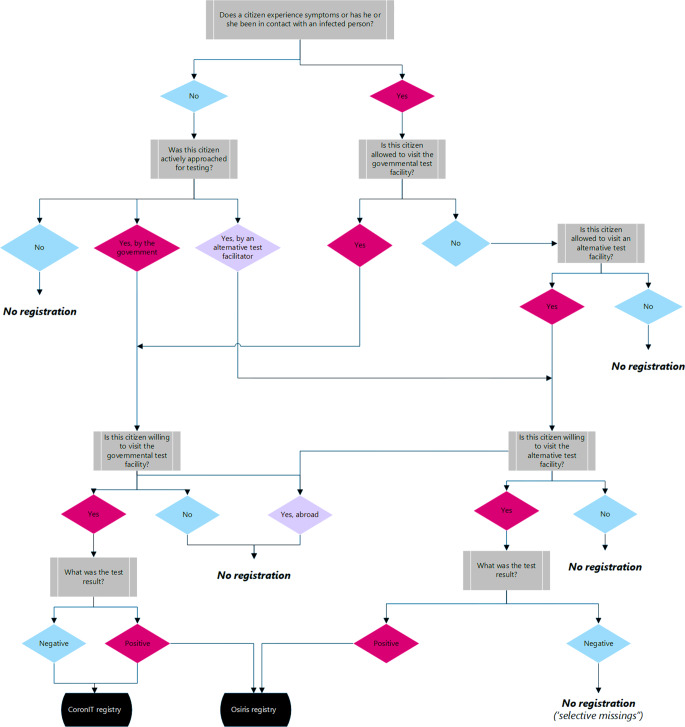
Schematic representation of interactions between test policy, individual compliance with test policy and registration policy and their influences on available registry data quality. ‘Alternative’ refers to all test locations other than governmental test facilities of the Dutch Municipal Health Services

The data quality of the COVID-19 registries was influenced by two aspects of public health policy. The first was the registration policy that stipulated which test results ended up in the official registries. The second aspect was the testing policy that stipulated who was eligible for a test at either the governmental test facilities or any of the alternative test locations. Test compliance of individuals was an additional complicating factor (a schematic representation is shown in Fig. 1).

The registration policy was unaltered during the study period. Citizens tested at the governmental test facilities were all registered in the CoronIT registration system, regardless of their test results. Those with a positive test result were also registered in the Osiris registration system, regardless of the test facility. As a result, negative test results of all citizens tested at alternative test facilities did not end up in the official registries. In addition, data from Dutch citizens tested across the border in Belgium were missing as there was no policy on the exchange of test results between Belgium and the Netherlands. In epidemiology, this type of missing values is referred to as ‘selective missing values’.

The testing policy was more complex and was changed over time. For example, the Dutch government strongly discouraged children to visit a test location at the start of our study period, but they were encouraged to do so in a later phase of the pandemic. Large-scale testing in nursing and care homes was part of the national policy advice at the start of the pandemic, when elderly were assumed to be the most vulnerable to SARS-CoV2 infection. In addition, citizens of specific districts or municipalities were actively approached for testing by their municipal health services, for example in response to a local outbreak or when low compliance with the test policy was suspected. Moreover, employers were made responsible for their own testing policy in the workspace. Therefore, testing policy is assumed to have varied largely between sectors and individual employers. As these local policies have not been documented in a structured way, their influence on the data quality for specific groups of employees is largely unknown.

In summary, the data quality for a population group is high if many citizens in this group were allowed to test at the governmental test facilities of their municipal health service and they were highly compliant with this testing policy. Our case study data showed an overrepresentation of members of households with children and with a high to very high household income derived from salary or shares at the governmental test facilities. If many citizens in a specific population group were not allowed to test at the governmental test facilities but only at the alternative test facilities according to the test policy, the data quality for this group is low. More specifically, the registry of positive test results in these groups is relatively complete, whereas the registry of negative test results is not. One method to identify population groups with a large proportion of missing negative test results is to study the proportion of positive test results detected at the alternative test facilities in each group. Our data showed that this proportion was the highest amongst citizens aged 65 years and over, those living in nursing homes or other care facilities and those with pension benefits as the main source of their household incomes. For population groups in which many citizens were not allowed to test at the governmental test facilities, were tested abroad or were not compliant with the test policy, the data quality is very poor. These groups can be considered as ‘data invisible’ as they could not be identified from the data of our case study. These examples illustrate how the complex interaction between test policy, registration policy and compliance with the test policy could have led to population differences in COVID-19 registry data quality.

## Potential effect of registration and testing policies on calculated infection rate

**Fig. 2:**
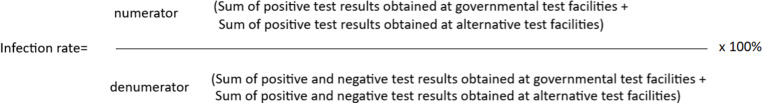
A formula for calculating the infection rate

Clearly, population differences in data quality lead to population differences in the reliability of calculated infection rates. The infection rate in each population group is often calculated according to the formula in fig. 2.

For population groups with good data quality, the calculated infection rate is quite reliable. For population groups with complete data for positive test results but not for negative test results, the calculated infection rate will always be an overestimation of the true infection rate. After all, the calculated denumerator will always be lower than the true denumerator. For population groups with very poor data quality, the infection rate cannot be calculated in a reliable manner. Only infection rates with similar reliability can be compared fairly. For example, in our case study, the infection rate could not be fairly compared between specific labour sectors nor between residents in private households and those in nursing homes due to these differences in data quality.

## Evaluation: implications for data-driven working in public health sector

However, these limitations do not imply that data-driven working is not possible at all. They rather substantiate the importance of comparable data quality. For example, differences in infection rates between different types of households within care institutions can be studied. After all, similar testing and registration policies applied to both types of households. As a result, the calculated denominator reflects a comparable underestimation of reality in both groups. By making proper comparisons, more reliable management information can be extracted. This reduces the risk of misinformation and can therefore eventually contribute to reduced stigmatisation of population groups. Decision-making based on simple geographical statistics, such as the infection rate at district level, is not recommended because of the abovementioned bias induced by testing and registration policies. Our case study shows that data quality can vary greatly within but also between districts.

## Future perspectives

We believe our case study provides a number of valuable insights. Existing legal frameworks, procedures and registration systems that worked well in regular practice of infectious disease control did not match the needs of a crisis as large as the COVID-19 pandemic. To better facilitate public health researchers to unravel indicators of true relevance for the third sustainable development goal of the United Nations—that is to ensure healthy lives and promote well-being for all at all ages—in the future, the following two steps are recommended.

### Recommendation 1: Invest in one reliable registration system that includes negative test results

Our advice is to invest in a single reliable registration system of positive and negative test results that is suitable for all possible test locations and for all possible infectious diseases. The use of such a system could avoid many of the population differences in data quality illustrated in our case study in the future. Besides more reliable estimates of population differences in infection rates, it would also provide valuable information about testing behaviour. Instead of mapping testing rate data obtained at test facilities of the Dutch Municipal Health Services only, testing rate data could be obtained from all inhabitants, regardless of their test location. That would provide an opportunity to calculate and visualise meaningful population differences in testing rates, estimate population differences in compliance with the test policy and develop population-specific public health interventions, in close collaboration with other countries.

### Recommendation 2: Build trust and involve citizens in improving data completeness

Compliance with the test policy is an important determinant of data completeness and quality. However, it is difficult to properly measure compliance on a full population level. In a future health crisis, we would not only benefit from more complete data but also from more varied data relevant for public health policy development. That is, data that can be directly used to substantiate the development of interventions, such as data on citizens’ motivations for either accepting or refusing a test or vaccination.

Our second recommendation is therefore to involve citizens more intensively in both data collection and public health policy development. Ideally, the future public health data architecture is flexible enough to ensure that citizens gain ownership of their public health data. It would be transparent and suitable for adding new data, and it would include an option for citizens to enrol in or withdraw from scientific studies at any moment. Having this kind of system would be an important step towards a more efficient approach to reduce health inequalities based on true citizen science.
